# Getting To Implementation (GTI)-Teach: A seven-step approach for teaching the fundamentals of implementation science

**DOI:** 10.1017/cts.2022.420

**Published:** 2022-06-17

**Authors:** Shari S. Rogal, Charles Jonassaint, LauraEllen Ashcraft, Janet Freburger, Vera Yakovchenko, Yasaswi Kislovskiy, Angela Phares, Gretchen Hershberger, David E. Goodrich, Matthew Chinman

**Affiliations:** 1 Departments of Medicine and Surgery, University of Pittsburgh, Pittsburgh, PA, USA; 2 Center for Health Equity Research and Promotion, VA Pittsburgh Healthcare System, Pittsburgh, PA, USA; 3 Department of Medicine, University of Pittsburgh, Pittsburgh, PA, USA; 4 Center for Health Promotion and Health Equity, Corporal Michael H. Crescenz VA Medical Center, Philadelphia, PA, USA; 5 Department of Physical Therapy, University of Pittsburgh, Pittsburgh, PA, USA; 6 Center for Healthcare Organization and Implementation, VA Bedford Healthcare, Boston, MA, USA; 7 Department of Obstetrics and Gynecology, Allegheny Health Network, Pittsburgh, PA, USA; 8 Center for Integrative Medicine, University of Pittsburgh Medical Center, Pittsburgh, PA, USA; 9 RAND Corporation, Pittsburgh, PA, USA

**Keywords:** Human-centered design, implementation science, healthcare, dissemination, translational science

## Abstract

**Introduction:**

Implementation Science (IS) is a complex and rapidly evolving discipline, posing challenges for educators. We developed, implemented, and evaluated a novel, pragmatic approach to teach IS.

**Methods:**

*Getting To Implementation* (GTI)-Teach was developed as a seven-step educational model to guide students through the process of developing, conducting, and sustaining an IS research project. During the four-week online course, students applied the steps to self-selected implementation problems. Students were invited to complete two online post-course surveys to assess course satisfaction and self-reported changes in IS knowledge and relevance of GTI-Teach Steps to their work. Results were summarized using descriptive statistics; self-reported post-course changes in IS knowledge were compared using paired t-tests.

**Results:**

GTI-Teach was developed to include seven Steps: 1. Define the implementation problem; 2. Conceptualize the problem; 3. Prioritize implementation barriers and facilitators; 4. Select and tailor implementation strategies; 5. Design an implementation study; 6. Evaluate implementation; 7. Sustain implementation. Thirteen students, ranging in experience from medical students to full professors, enrolled in and completed the first GTI-Teach course. Of the seven students (54%) completing an end-of course survey, six (86%) were *very satisfied* with the course. Ten students (77%) responded to the tailored, 6-month post-course follow-up survey. They retrospectively reported a significant increase in their knowledge across all steps of GTI-Teach (1.3–1.8 points on a 5-point Likert scale) and rated each of the Steps as highly relevant to their work.

**Conclusions:**

GTI-Teach is a seven-step model for teaching IS fundamentals that students reported increased their knowledge and was relevant to their work.

## Introduction

Implementation Science (IS) is a relatively new translational science discipline that focuses on strategies to adopt, integrate, and spread evidence-based practices and interventions [1,2]. IS approaches have been applied across a range of fields, such as agriculture, education, and health [3,4]. In translational health sciences research settings like Clinical Translational Research Award (CTSA) programs, IS methods and strategies are increasingly seen as valuable for accelerating knowledge translation within and across all phases of the research continuum [2,5,6]. Subsequently, there is keen interest in supporting IS training and educational offerings [7–10].

IS experts have identified certain challenges in teaching core IS competencies and principles to researchers and practitioners [10–13]. Because IS has a broad orientation that reflects its eclectic origins in a number of disciplines (e.g., agriculture, public health, organizational theory), acquiring competency in the field requires a wide range of knowledge and skills. IS trainees must integrate a broad number of theories, models, and frameworks; contextual assessment; evaluation designs; methods; measurement; analysis; and novel regulatory issues (e.g., understanding when IS is conducted as research versus quality improvement or when participant informed consent is or is not required) [10]. Additionally, trainees must maintain a connection to the clinical, translational science, or public health area where they are applying IS methods, requiring them to straddle both IS and their topic area.

While the broad and rapidly expanding knowledge base related to IS can be inaccessible or overwhelming to the uninitiated, there are several cross-cutting principles and competencies that IS experts agree are fundamental to master, regardless of a learner’s role, context, or aim for applying IS [2,4,10,11,14,15]. We aimed to embed these requisite skills and theories in an overall coherent IS educational approach to make IS training more accessible and practical, while also incorporating adult learning principles [16]. Thus, we started with *Getting To Implementation* (GTI), a manualized implementation support approach for health care settings to select, tailor, and apply implementation strategies to improve the quality of health care [17].

GTI is an example of a “process framework,” or steps to guide researchers, practitioners, and policy makers through the process of translating evidence into practice. It is based on *Getting To Outcomes* (GTO)®, an evidence-based, 10-step implementation support model and toolkit that has been manualized and designed to assist community organizations in selecting, tailoring, implementing, and evaluating evidence-based programs and interventions [17–25]. When asked to develop an IS introductory course, GTI developers felt that, with small adaptations, it could be a useful teaching approach, given its early success in transferring knowledge to clinicians enrolled in a GTI trial [17]. Thus, authors SR, MC, CJ, JF, and LEA collaborated to adapt GTI to GTI-Teach, a seven-step approach to help learners understand and execute core IS competencies in research and community-engaged practice projects. This report describes how we developed and piloted this training course and assessed learning outcomes.

## Methods

### Target Audience and Online Format

This course was offered through the University of Pittsburgh’s Institute for Clinical Research and Education (ICRE) within the Clinical and Translational Science Institute (CTSI), and was intended for students, trainees, and faculty from across the University seeking an introduction to IS, particularly those interested in implementation within large health systems. The course was advertised through the Institute of Clinical Research Education (ICRE) website and discussed during meetings of the University of Pittsburgh’s Dissemination and Implementation Science Collaborative (Pitt DISC). Participants registered in advance for the course, which was held over a 4-week period in March–April 2021 and allowed registration of up to 15 participants. Though the class was intended to be delivered in-person, pandemic accommodations necessitated pivoting to an online format.

The course was taught synchronously online and was recorded, such that students could watch the course recording asynchronously if they could not attend. The course was taught as a mixture of didactic lectures and interactive sessions. Students could pose questions in the chat or during discussions during and after each lecture. Students were encouraged to participate with direct questions asked during the lectures. As students worked through their own slides and projects, instructors (n = 3) and other students provided direct feedback in breakout sessions (using breakout rooms).

### Educational Methods and Curricular Program

The principles and learning objectives for this course were intended to develop core compentencies in IS and to enable learners to apply these concepts and methods to their own work. Thus, to further enhance learning in a pragmatic way, students were asked to actively apply the GTI framework to an evidence-to-practice gap that was germane to their area of research. Students engaged in a concrete and sequential process of developing an IS research project, starting with defining an implementation problem (i.e., evidence-to-practice gap) and conceptualizing the project to an implementation trial and sustainment of the evidence-based practice.

### GTI-Teach Development

**Fig. 1.**
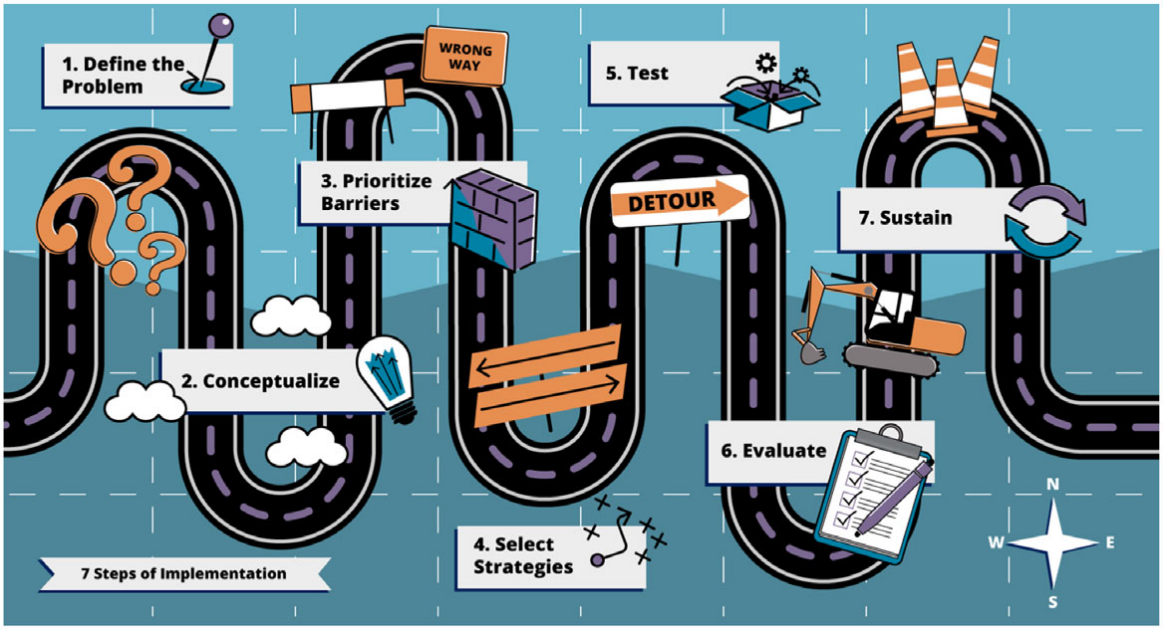
GTI-Teach steps.

GTI-Teach was designed as a step-by-step approach for educators and students based on GTO and GTI. Table 1 compares the steps of GTO, GTI, and GTI-Teach. The steps of GTI-Teach were discussed and iterated by five content experts (SR, MC, CJ, JF, and LEA) over a 3-month period until consensus was reached on the order and content of GTI-Teach steps. GTO is a 10-step process that was previously adapted as a tool to guide healthcare settings to select and apply implementation strategies. Two fundamental changes were required to adapt GTI into a teaching tool. First, GTI is intended to be used by frontline providers, but IS students require a larger focus on the frameworks, models, and theories which underlie IS, and methods for understanding the mechanisms underlying their target behavior(s). Thus, we added GTI-Teach Step 2 – conceptualizing implementation. Conversely, the later GTI steps focus on the work of implementation and iteration, so GTI-Teach was changed to focus on understanding the general concepts and frameworks underlying implementation evaluation and sustainment, two major areas of IS that are also critical to successful implementation. These areas are covered in the GTI-Teach step 6, evaluate implementation, and 7, sustain implementation. GTI-Teach was thus conceived as a seven-step process (Fig. 1). These “steps” were used to develop the class syllabus. A templated slide deck was developed to guide learners through the GTI-Teach steps (Supplemental File 1) as a way of developing an IS project that applied to their area of study or discipline.

**Table 1.** Steps of Getting to Outcomes (GTO) and its evolution to GTI and GTI-Teach

GTO	GTI	GTI-Teach
1. Needs and resources assessment	1. Develop team and assess current processes	1. Define the implementation problem
2. Goals and desired outcomes	2. Establish goals	
--	--	2. Conceptualize the problem
3. Fit	3. Assess and prioritize strengths and barriers	3. Prioritize implementation barriers and facilitators
4. Capacities		
5. Select best practices	4. Choose strategies	4. Select and tailor implementation strategies
6. Plan	5. Plan and adapt strategies	5. Design implementation study
7. Process evaluation	6. Implement, evaluate, and improve	6. Evaluate implementation
8. Outcome evaluation		
9. Continuous quality improvement		
10. Sustainment	7. Sustain implementation	7. Sustain implementation

GTO, Getting to Outcomes; GTI, Getting to Implementation.

### Mapping IS Competencies to the Steps of GTI-Teach

Table 2 illustrates how IS competencies align with the GTI-Teach steps. Competencies for beginner and intermediate levels were included in our mapping with the expectation that the students started the class with a diverse range of baseline knowledge. The depth with which each topic was covered was tailored to the students’ levels. For example, one student had large-scale implementation science funding, so the methods that were of interest to that student were distinct from the students with no prior experience. Five advanced skill competency topics were acknowledged but not covered in sufficient detail to develop student proficiency (e.g., de-implementation, tailoring implementation strategies).

**Table 2.** GTI-Teach and IS competencies

GTI-Teach	IS competencies covered in GTI (adapted from Padek et al [15])
Define the implementation problem	• Understand IS research terminology (B) • Define what is versus what is *not* IS (B) • Differentiate between IS research and related disciplines (B) • Identify the potential impact of IS work (B) • Describe the range of IS methods (B) • Identify EBPs worth implementing (I) • Explain de-implementation in IS (A)
Conceptualize the problem	• Describe a range of IS strategies, models, and frameworks (B) • Evaluate context in IS (I)
Prioritize implementation barriers and facilitators	• Describe the importance of incorporating partners (B) and organizational partner perspectives in IS (A) • Describe participatory methods (B), how to apply them to IS (I) • Apply common IS measures and analytic strategies (I) • Measure partnerships for IS research (I)
Select and tailor implementation strategies	• Evaluate and refine implementation strategies (A) • Identify a process for adapting an intervention in IS research (I) • Explain fidelity and adaptation when developing interventions/strategies (I) • Tailor strategies (A)
Design implementation study	• Describe study designs for IS research (I) • Identify and recruit sites for IS research (I) • Integrate mixed methods in IS research (I)
Evaluate implementation	• Identify IS measures (B) • Work with partners to select IS outcome measures (I) • Incorporate economic evaluation into IS work (A) • Describe the concept and measurement of fidelity (B)
Sustain implementation	• Integrate sustainability plans and concepts into work (I) • Develop sustainable IS partnerships (I)

IS, implementation science; GTI, Getting to Implementation; EBP, Evidence-based practice; Competency is mapped to skill level such that (B), Beginner to IS; (I), Intermediate; and (A), Advanced.

### Embedding Health Equity and Human-Centered Design Throughout the Course

The planning group decided *a priori* to emphasize health equity and human-centered design, based on emerging literature regarding their importance in IS and the expertise of the instructors [7,26–31]. As such, we presented a lecture by a health equity expert early in the course and illustrated the Health Equity Implementation Framework [32] as an example of a key IS Framework. We then discussed equity as a crucial aspect of proactive planning and tailoring of the evidence-based practice and implementation strategies for known disparities and barriers (determinants) in priority populations and implementation contexts. Moreover, we emphasized the importance of iterative and ongoing measurement and evaluation of health equity over time as an essential implementation outcome that reflects the quality of sustainability capacity to adapt the “fit” of the evidence-based practice to dynamic context. In our course evaluation, we evaluated the extent to which students felt we had addressed equity.

Human-centered design (HCD) is a philosophy and associated set of techniques to uncover unmet patient needs, promote community and organization partners buy-in, and build trust [33,34]. In the first lecture, students were introduced to Mural (Tactivos, Inc), a software tool that enables visualization of ideas for collaborative problem-solving and innovation. Throughout the course, examples of HCD activities were presented as complementary methods to IS to not only engage community and organization partners in the co-production process of developing implementation interventions, but to also make course content practical and relevant to students. Specifically, HCD enhances GTI-Teach by enabling students to experience how powerful visual thinking activities can enable interdisciplinary teams to sequentially approach the design of complex innovations (e.g., evidence-based practices, implementation strategies) that are desirable, feasible, and viable for intended users.

**Fig. 2.**
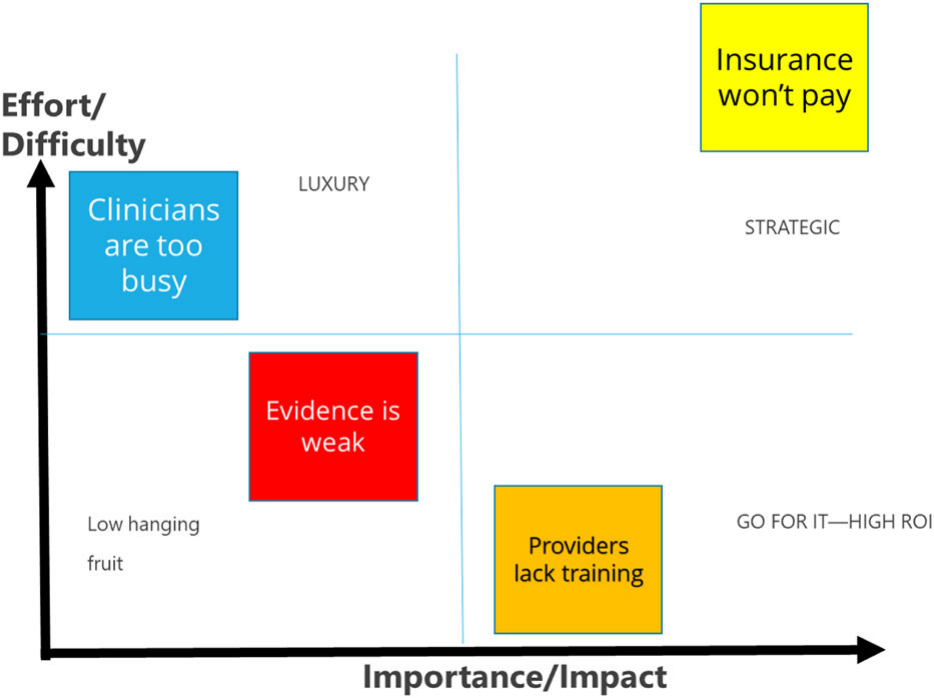
Human-centered design example: importance-difficulty matrix used to prioritize implementation barriers. ROI, Return on investment.

Paralleling the steps of many IS process frameworks and methods (e.g., Implementation Mapping [35]), HCD methods originally developed in engineering and business contexts are useful to harness the creativity of collaborative teams to generate ideas, insights, and options into a health care problem through divergent thinking activities (e.g., brainstorming, affinity diagrams) before using convergent thinking activities to help teams synthesize, analyze, and eliminate options to generate solutions that can be rapidly prototyped for iterative testing [36]. Similarly, the outcomes used by IS to assess the effectiveness of implementation processes (e.g., feasibility, acceptability, appropriateness [37]) are easy to map to the HCD qualities of a well-designed health care innovation for frontline users (e.g., simple, fit, efficient, learnable, satisfying) [29]. For example, students were introduced to an importance-difficulty matrix, where they could work with community partners to rate the relative importance of barriers to implementing an evidence-based practice and then the perceived difficulty of addressing these barriers as an approach to prioritizing implementation barriers (Fig. 2) [38].

### Course Materials

Materials, including readings, slides, and summary documents were available on a shared drive for learners. Each step in GTI-Teach was assigned a graphical representation (Fig. 1) and the theme of the steps was carried through interactive didactic sessions, lecture slides, and templated homework and presentations. Supplemental File 1 includes a blank slide template that students completed with the aid of information learned in the class. An asynchronous video was also produced to illustrate the steps (though this was made available after the course ended due to limitations with production capacity). See: https://www.youtube.com/watch?v=y6zhwTvwFsU&t=5s

### Adult Learning Principles

We adhered to adult learning principles as we built GTI-Teach and the classroom environment [16]. For instance, such theory posits that adults bring experiences to the classroom, so we focused on establishing a classroom environment wherein we were co-learning with students and participants were invited to provide examples of course concepts from their experiences. Students were also treated as co-facilitators and invited to provide feedback to peers. Thus, the steps were geared toward connecting their life and prior experiences to the new material. Because adult learners are goal-directed, we created clearly defined elements and demonstrated how the findings could be used in their current work. Finally, we encouraged group learning and a variety of options for learning the material, including online lectures, recorded videos of class, readings, discussions, participatory activities, and guest lectures.

### Course Implementation

The course was conducted virtually on the institutional Zoom platform (Zoom Video Communications, Inc.) over a 4-week period, with two 2-hour classes per week. The course was led by three primary instructors (SR, CJ, and JF) who participated in nearly all classes and small group sessions. The 2-hour sessions were structured as four 25- to 30-minute segments. The first segment was a didactic session introducing an IS concept relevant to the corresponding GTI-Teach step for that lesson. The next segment was a small group activity of four to five students and one instructor, to help solidify the concept or discussion about the topic and how it relates to student projects. The third segment typically involved another lecture introducing the homework assignment and conveying expectations for completing the template slide. Finally, the last segment involved small groups to enable each learner to start working on their homework assignment and receive feedback from their peers and one of the instructors.

All classes concluded with a summary of what was covered, addressed any final comments or answer remaining questions, and provided expectations for the next class. Projects were expected to follow the slide template, with one to three slides per step. Classes introduced several theories, models and frameworks that are commonly employed by implementation scientists while guest lecturers presented on topics including engaging community and organization partners, health equity, and Diffusion of Innovations Theory [39] as an opportunity to understand the concepts of passive versus active dissemination of innovations as well as why individuals and organizations adopt new innovations (see Supplemental File 2 for syllabus).

### Course Evaluation Surveys

The evaluation included two post-course surveys: an end-of-course survey and a 6-month post-course follow-up survey. The first, an “end-of-course survey” was emailed to students on the last day of the class by administrators of the University of Pittsburgh’s Institute of Clinical Research Education (ICRE), with two subsequent reminder emails. This generic, cross-course, 14-item “end-of-course course survey” was developed by ICRE’s educational experts for participants in the University’s Clinical and Translational Science Institute (CTSI) training and educational programs. This survey asked students to rate (on 5-point Likert scales) their degree of agreement or satisfaction pertaining to nine aspects of the course including: clarity of goals and objectives; readings; course content; assignments; respect for diversity; use of inclusive materials; instructor availability; and use of Zoom sessions. Three items used a 3-item Likert-type format for students to rate course workload, pace, and assignment difficulty while two open-ended questions elicited comments on positive aspects of the course as well as suggestions for improvement.

The second survey (Supplemental File 3), a 10-item “6-month post-course follow-up survey” was developed de novo by the course instructors to ask tailored questions about the content specific to GTI-Teach. This second post-course survey was approved by the University of Pittsburgh IRB as an exempt study. Students were asked to think back upon their pre-course knowledge and rate (on 5-point Likert-type scales) knowledge about each of the GTI-Teach Steps/topics *before the course* and *after the course*. They were also asked about the usefulness of each Step/topic in their current work. Other questions requested feedback on specific aspects of the course such as the incorporation of user-centered design principles and tools, guest lecturers, slide templates, and specific ways students applied course knowledge after the course (e.g., to grant writing). Open-ended comments and suggestions were also requested on this survey using a free text box.

### Survey Analysis

Summary statistics (e.g., means, medians) were used to describe the responses to the two post-course surveys. The retrospective change in knowledge questions on the 6-month, post-course follow-up survey were analyzed using paired t-tests.

## Results

### Course Participation and Completion

Thirteen students enrolled in and completed the 4-week course. Students ranged in experience from medical students to senior research scientists and included four men and nine women. Faculty and students from the University of Pittsburgh’s Schools of Dentistry, Public Health, Medicine, Social Work, Rehab Sciences, and Pharmacy attended. All classes were virtual and led by the core instructors (SR, CJ, and JF) with intermittent guest lectures. All students completed templated oral presentations at the end of the 4-week period, applying the seven GTI-Teach Steps to develop a real-world study of their own design. These presentations were largely related to health and included diverse evidence-based practices in dentistry, HIV prevention, autism treatment, and medical transport.

### End-of-Course Survey

Seven of thirteen students (54%) completed an electronic generic end-of-course survey used across ICRE courses to assess the appropriateness and satisfaction with course delivery and content. As summarized in Table 3, all respondents to the post-course survey agreed that the course content met their needs, that the assignments were “*just right*” in terms of difficulty, that the goals and objectives were clear, and that the reading enhanced their learning. All students were at least moderately satisfied with the online format. However, 86% of students (6 of 7 respondents) felt the pace was “*too fast*.”

**Table 3.** End-of-course survey results (n = 7)

Question	Rating scale (n, %)			Mean ± SD (on 5-point Likert scale)
	Neither	Agree	Strongly agree	
Goals and objectives of course were clear*		4 (57%)	3 (43%)	4.4 ± 0.5
Text/reading is enhancing my learning*	1 (14%)	4 (57%)	2 (29%)	4.1 ± 0.7
Course content is appropriate for my needs*	1 (14%)		6 (86%)	4.7 ± 0.8
Assignments reinforced the learning goals*			7 (100%)	5.0 ± 0
Instructor respected cultural & personal differences*			7 (100%)	5.0 ± 0
Instructor included inclusive learning materials*			7 (100%)	5.0 ± 0
Amount of interaction with instructor met needs*			7 (100%)	5 ± 0
Zoom sessions enhanced learning*			7 (100%)	5 ± 0
	**Too little**	**Just right**	**Too much**	
Course workload is appropriate**		6 (86%)	1 (14%)	
Assignments were appropriate level of difficulty**		7 (100%)		
	**Too slow**	**Just right**	**Too fast**	
Course pace is appropriate**		1 (14%)	6 (86%)	
	**Neutral**	**Moderately satisfied**	**Very satisfied**	
Satisfaction with fully on online format^†, ‡^		1 (14%)	6 (86%)	

* Items were rated on 5-point Likert scale ranging from 1=*Strongly Disagree* to 5=S*trongly Agree*.

** Item(s) rated on a 3-point scale.

† Course was delivered fully online due to Covid pandemic restrictions.

‡ Item was rated on 5-point Likert scale ranging from 1=*Very satisfied*, 3=*Neutral*, to 5=*Very satisfied*.

### Six-Month Post-Course Follow-Up Survey

In addition to the generic end-of-course survey, ten students (77%) responded to the tailored, course-specific, IRB-approved, 6-month post-course survey, which asked students to reflect on the extent to which the GTI-Teach Steps increased their self-reported knowledge and were relevant to their work (Table 4). When asked “To what extent was the 7-step method a helpful education tool,” on a 5-point Likert scale ranging from one (*not helpful*) to five (*extremely helpful*), all respondents rated the GTI-Teach to be helpful, with half rating it “*very helpful*” and the other half rating it “*extremely helpful*.” Students similarly rated the slide templates as *very* (40%) or *extremely* (60%) helpful in applying content to their self-selected project.

**Table 4.** Six-month post-course follow-up survey results (n = 10)

GTI-Teach step	Topic covered	Baseline knowledge IS topics*, Mean ± SD (5-point Likert scale self-rating	Post-course knowledge IS topics*, Mean ± SD (5-point Likert scale self-rating	Change in knowledge, Mean ± SD (change between two 5-point Likert scale ratings)	How useful IS topics are to work^†^, Mean ± SD (5-point Likert scale self-rating
1	Defining an implementation problem	2.3 ± 1.0	3.7 ± 6.6	1.4**	4.2 ± 1.0
2	Conceptualizing an implementation problem	2.1 ± 1.0	3.6 ± 0.8	1.5**	4.3 ± 1.0
3	Evaluating barriers to implementation	2.1 ± 0.9	3.6 ± 0.5	1.5**	4.3 ± 0.9
4a	Selecting implementation strategies to overcome the barriers	1.4 ± 0.7	3.2 ± 0.6	1.8**	4.3 ± 0.9
4b	Tailoring implementation strategies to the context	1.7 ± 1.0	3 ± 0.6	1.3**	4.3 ± 0.9
5	Designing an implementation study	1.7 ± 0.6	3.5 ± 0.8	1.8**	4.2 ± 1.0
6	Evaluate implementation	1.9 ± 0.7	3.2 ± 0.9	1.3**	4.3 ± 0.9
7	Sustaining implementation	1.4 ± 0.5	3.0 ± 0.6	1.6**	4.3 ± 1.0
	Overarching principle: Considering health equity in implementation science	1.6 ± 0.5	3.0 ± 0.8	1.4**	4.5 ± 0.9

GTI, Getting to Implementation; IS, implementation science.

* Knowledge rated on 5-point Likert sale, 1=*Not knowledgeable* to 5=*Extremely knowledgeable*.

** p < 0.001.

† Usefulness rated on 5-point Likert scale, 1=*Not useful* to 5=*Extremely useful*; IS, implementation science.

Two questions asked students to rate their perceived change in knowledge for each of the GTI-Teach steps and considering health equity from “coming into the course” to “completing the course.” The stem for each question asked “To what extent did you feel knowledgeable about each of the following topics” with respondents rating on a 5-point Likert scale ranging from one (*not knowledgeable*) to five (*extremely knowledgeable*). The average improvement within each content area was +1.3 to 1.8, with significant change in perceived knowledge for each topic area. Students were also asked how they had applied the IS training over the 6-month period after the class. Five students (50%) reported that they had applied the conceptual frameworks to their research work and four (40%) reported using what they learned in the course to aid in grant development.

### Feedback about Incorporating Human-Centered Design Tools into the Course

Students disagreed about the ideal extent to which human-centered design tools should be incorporated into the course: 44% (n = 4) thought there was the right amount of tool use; and two reported too much and two reported too little was included. There were several technical challenges with navigating the Mural software platform. As such 44% (n = 4) participants liked the interface, while 66% (n = 5) felt the platform was used too much.

### Course Adaptations

The following fidelity-consistent adaptations were made to the syllabus and teaching methods to address feedback from students and instructors as outlined in Table 5. Where applicable, we include supportive quotes from open-ended questions from the end-of-course and post-course follow-up surveys.

**Table 5.** Adaptations to GTI-Teach based on student feedback

Done to address	Adaptation type [40]	Supportive quote or data	Operationalization
Fast pace	Pacing decreased	*“For me, there was too much information in too little time.”*	One step per week Develop videos to support asynchronous learning
Confusion over multiple models and frameworks	Decrease content in the conceptualize step	*“I found the pieces about COM-B [IS theory] to be a bit confusing and hard to integrate into the other taxonomies.”*	Focus on two meta-frameworks rather than many frameworks, models, and theories
Conceptually distinct steps were combined in a single step	Content: Spreading	*“Addressed the pacing issue described above.”*	Separate former step 6 into designing the trial/test and evaluate
Student request about grading/equity	Format	*“I had trouble finding time to do the readings outside of class. But that has nothing to do with the class.”*	Course was made pass fail and readings optional

COM-B, an implementation science theory; IS, implementation science.

## Discussion

GTI-Teach was developed to introduce IS fundamental concepts to a wide range of learners in a short timeframe. Given its connection to existing IS frameworks and practical focus, GTI-Teach fills an important gap among IS educational offerings by introducing IS to a broad audience of interdisciplinary researchers and practitioners using a simple, stepwise approach.

Prior publications have advocated for IS training. In one survey of 37 CTSAs, 63% reported that IS training was important and 33% advocated for coordination across hubs in such training activities [1]. Other IS leaders have similarly advocated for introductory courses that provide foundational principles relevant to interdisciplinary trainees [10,14]. GTI-Teach aligned well with expert recommendations about IS competencies [10,12,14,41–44]. We focused on covering the majority of beginner and intermediate topics, providing an overview rather than a deep dive into advanced topics of design, evaluation, and analysis for learners who were predominantly new to IS. GTI-Teach also encouraged implementation *researchers* (i.e., those who will advance IS models, strategies, and methods) and implementation *practitioners* (i.e., those who will use models, strategies, and methods) to co-learn together to ensuring that all learners have a common language and core understanding of key principles in the IS field which strengthens the IS capacity of the local CTSA workforce [14].

There are several challenges in designing introductory IS courses. First, IS is a rapidly evolving field. Second, IS knowledge and expertise must be acquired in addition to a learner’s primary discipline [10]. To address this complexity, IS principles must be accessible to non-implementation scientists across disciplines. For example, there is a movement toward considering implementation and design early in intervention development and scientific inquiry [2]. IS experts have proposed that pre-clinical researchers receive IS training to learn how to accelerate the research process [2,6,14]. The COVID pandemic has highlighted the need to think proactively about systems and health equity challenges [45]. Reporting on training and capacity-building initiatives such as GTI-Teach can decrease redundant efforts and address gaps in expertise and the need for a larger IS workforce to meet the needs of communities and academic intitutions [46,47].

This evaluation and course were not designed to answer the question of optimal teaching format for the transfer of IS knowledge (e.g., synchronous online, face-to-face workshops, academic courses). However, there is clearly a need to make content transparent and accessible to a variety of community members. Developing local IS training capacity can be difficult in institutions that lack resources, faculty, or research leadership understanding of IS value [10,12]. Some CTSAs have therefore created regional collaboratives to repurpose national curricula (i.e., TIDIRC – the National Cancer Institute’s *Training Institute for Dissemination and Implementation Research in Cancer*, a 6-month facilitated training course facilitated by IS experts with competition for a fixed number of learning spots, or the open-access version of TIDIRC made available after the facilitated institute), expert video presentations, and pooled faculty to create capacity [44,48]. The GTI-Teach approach aims to be quick to learn, simple, practical, and applicable to those applying for grant applications and new learners without a tremendous time investment.

Next steps for the University of Pittsburgh’s CTSI will include exploring micro-credentialing in IS and developing a track in an existing health services Master’s program that focuses on IS. Micro-credentialing is a practical strategy that offers learners the opportunity to obtain a graduate certificate focused on dissemination and implementation science competencies from an accredited academic institution rather than pursuing a full graduate degree [14]. However, because traditional educational programs remain limited in their capacity, we are currently adding asynchronous online, accessible content for those new to the field.

This approach is not without limitations. First, the small sample of trainees, absence of pre-course, baseline assessments, and reliance of retrospective self-report of knowledge pre- and post-course provide only very preliminary data on the usefulness of this approach. Second, this first round of GTI-Teach included multiple faculty and guest lecturers; this approach may not be replicable at institutions that lack faculty and expertise in IS. Third, the 6-month post-course follow-up survey asked about the elements of the course and was not a standardized, valudated instrument. This allowed us to get practical feedback but was not as rigorous as using validated educational assessments of knowledge. Additionally, because the surveys were anonymous, they cannot be linked to each other or evaluated for systematic differences between responding and non-responding students. A final limitation is the lack of standardized language/terminology and single, consensus competencies across disciplines that use IS, which hampers development of simple educational materials and risks redundancies and inconsistencies [10,11].

## Conclusions

We successfully developed a new curriculum to teach foundational IS principles to a wide range of learners. GTI-Teach incorporates the pragmatism of simple, step-wise process frameworks to address complex IS competences. Designed to apply to across disciplines, GTI-Teach students from diverse departments reported significant increases in their knowledge from this 4-week, eight-session course.

## Supplementary material

For supplementary material accompanying this paper visit https://doi.org/10.1017/cts.2022.420. click here to view supplementary material
