# Supplementary material for: Getting To Implementation (GTI)-Teach: A seven-step approach for teaching the fundamentals of implementation science
Source: J Clin Transl Sci. 2022 Jun 17;6(1):e100. doi: 10.1017/cts.2022.420 (PMC9428668; doi:10.1017/cts.2022.420)
Supplement: Supplementary file 1 [file ctssup.zip › S2059866122004204sup002.pdf]

INSTITUTE FOR CLINICAL RESEARCH EDUCATION

Pittsburgh DISC

GTI-Teach Seven-Step Template

1. Define the Problem

- Background: What is the health problem of interest?
- Problem Statement: What is the implementation gap you are addressing?

Frame the problem in behavioral terms: who needs to change what to improve outcomes?

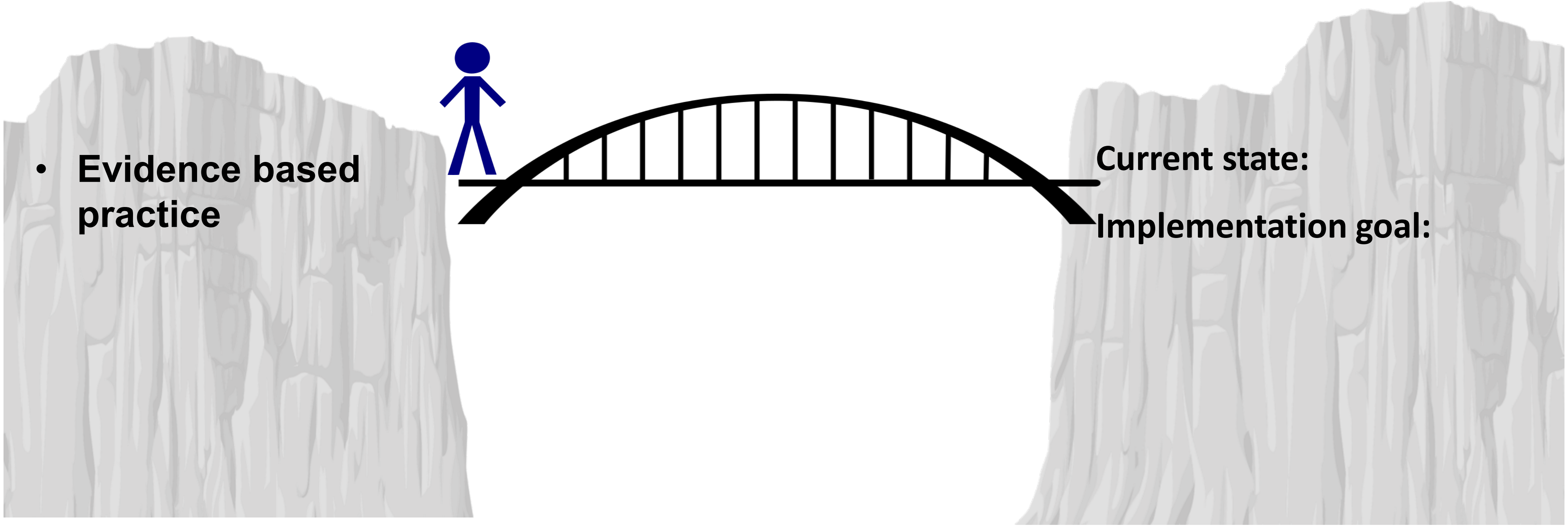

2. Conceptualize the Problem

2 Conceptualize

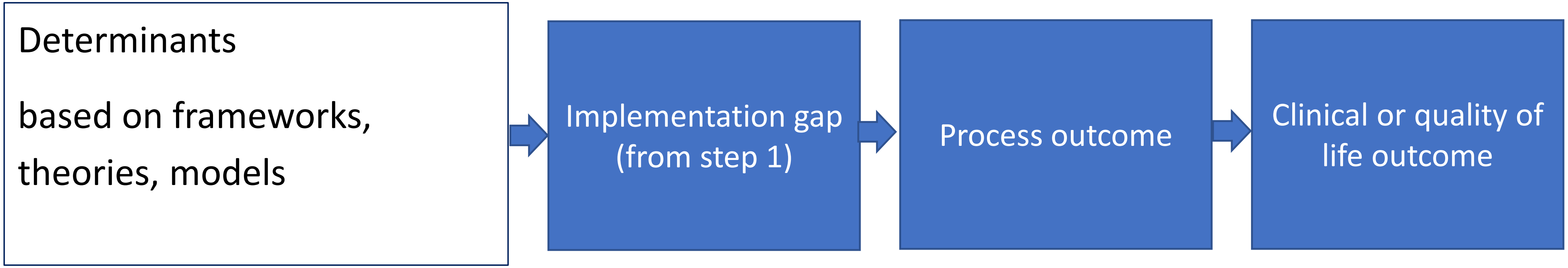

3. Prioritize Barriers (also Facilitators!)

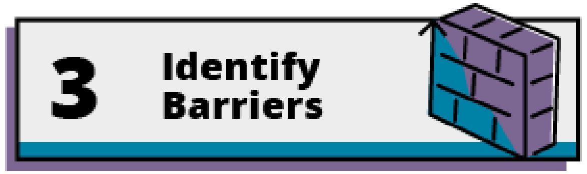

Methods:

1. Who are your stakeholders?

2. How will you engage them in refining your conceptual model and prioritizing barriers?

What are the barriers your implementation strategies will address?

4. Select & specify strategies

4 Select Strategies

Strategy Component	Strategy 1	Strategy 2
**Operationalize it**		
Who—actor who delivers strategy		
The action		
The action target		
Temporality		
Dose		
Implementation outcome		
Justification—what barrier is this addressing?		

5. Test Implementation

- Trial design:
  - To what extent will you focus on implementation vs. effectiveness?
  - What are your comparators? (one strategy vs. control or another strategy)
  - What is the set-up of the trial (e.g., stepped-wedge, cluster-randomized)
  - Level of randomization and analysis (clinic, clinician, patient)
- Setting:
- Inclusion criteria (clinics, clinicians, patients etc…)
- What is your primary outcome?
- When will you evaluate outcomes?
- Justification for choices:

6. Evaluate

6 Evaluate

- Which evaluation framework will you use?
- Which implementation outcomes will you focus on? (hint: consider stakeholders' opinions)
- Operationalize each implementation outcome, in terms of definition, timing, method of measuring

Example Outcome	Example measure
Reach	How many patients got the EBP? Was the EBP delivered equitably?
Effectiveness	To what extent did the EBP influence clinical outcomes?
Adoption	To what extent did clinics Adopt the EBP (e.g., deliver to a threshold % of patients)
Implementation	
Fidelity	To what extent was the EBP and strategy done as intended?<br>Fidelity checklists for strategy and intervention
Acceptability	To what extent was the strategy acceptable? To what extent was the EBP acceptable?
Cost	How much does this cost to do? Micro-costing the strategy
Sustainment	Recheck all measures at 6 months post intervention

7. Sustain

Questions	Answers	Next Steps (Explain or enter "N/A")
Which results can we use to justify ongoing implementation?		
What should we change about the way we implement?		
Who will support and lead sustainability efforts?		
How will we monitor key implementation outcomes in a sustainable way?		
How much funding, if any, do we need?		
How can we keep staff trained?		
