# Supplementary material for: Getting To Implementation (GTI)-Teach: A seven-step approach for teaching the fundamentals of implementation science
Source: J Clin Transl Sci. 2022 Jun 17;6(1):e100. doi: 10.1017/cts.2022.420 (PMC9428668; doi:10.1017/cts.2022.420)
Supplement: Supplementary file 1 [file ctssup.zip › S2059866122004204sup001.docx]

**Supplemental File 2. Syllabus**

| **Session** | **Learning Objectives/Topics** | **Example topics** |
| --- | --- | --- |
| 1  What is Implementation Science | 1. Understand whether you have an implementation question 2. Conduct an evidence-practice gap analysis (Step 1) | - The need for implementation science - IS across the translation research continuum - What IS is and is not - Implementation Outcomes framework - Introduction to GTI - Example IS projects - Example evidence-practice gaps |
| 2  Theories, Frameworks, and Models (TFMs) | 1. Understand the role of community and organizational partners and participatory methods in IS 2. Describe basic TFMs in IS 3. Conceptualize the problem (Step 2) | - Identify your implementation science question with the “who” and “what” - Intro to IS theories (organizational culture, implementation climate) - How to select a target behavior - Select your target behavior activity - Identify your appropriate patient, organizational, and community partners - Step 1 & partnership plans |
| 3.TFMs continued | 1. Understand the difference between Theories, Process Models, Determinant Frameworks and Evaluation Frameworks | - Define frameworks, theories, and models (TFMs) - Activity: Working with the FTMs definitions; Step 2 |
| 4  Frameworks, Theories and Models in the Real World:  Health Equity | 1. Entry-level understanding of how theories, models, and frameworks work together to answer implementation problems 2. Prioritizing barriers and facilitators (Step 3) | - Discusses the utility of frameworks, theories, and models in IS - Discuss the application of theory to behavior, social relationships, and how people interact - Determinant frameworks - Complete Step 3 |
| 5  Implementation Strategies | 1. Understand how to find the appropriate strategy to match your target behavior (Step 4) | - Selecting and specifying implementation strategies - Step 4 |
| 6  Designing and evaluating Implementation trials | 1. Choose and use an appropriate study design evaluation framework (Steps 5-6) | - Evaluation Frameworks and measures - Choose your framework and design Workshop - Activity: RE-AIM |
| 7. Adaptation, fidelity, and sustainment | Measure implementation and consider sustainment (Steps 6, 7) | - Adaptation and Intervention Fidelity - Documentation, Evaluation, Sustainment |
| 8. Final Presentations | Students present 7 step templates and give and receive feedback | - Present final project |

GTI= Getting to Implementation; IS = Implementation Science; FTM = Framework, Theories, and Models;

**Supplemental File 3. Six-month Post-Course Follow-up Survey**

1. To what extent was the 7-step method a helpful education tool?

| Not helpful  (1) | Slightly helpful  (2) | Moderately helpful (3) | Very helpful  (4) | Extremely helpful (5) |
| --- | --- | --- | --- | --- |
|  |  |  |  |  |

1. Do you think that selecting and tailoring implementation strategies can be combined?

- Yes
- No

1. To what extent do you think that the slide templates were helpful?

| Not helpful  (1) | Slightly helpful  (2) | Moderately helpful (3) | Very helpful  (4) | Extremely helpful (5) |
| --- | --- | --- | --- | --- |
|  |  |  |  |  |

1. To what extent did you feel knowledgeable about each of the following topics prior to and at the end of the course?

| **Implementation Science Topic** | 1=Not knowledgeable  2=Slightly knowledgeable  3=Moderately knowledgeable  4=Very knowledgeable  5=Extremely knowledgeable | |
| --- | --- | --- |
|  | **Coming into the course** | **After leaving**  **the course** |
| 1. Define an implementation problem |  |  |
| 1. Conceptualize an implementation problem |  |  |
| 1. Evaluate barriers to implementation |  |  |
| 1. Select implementation strategies to overcome the barriers |  |  |
| 1. Tailor implementation strategies to the context |  |  |
| 1. Design an implementation study |  |  |
| 1. Evaluate implementation |  |  |
| 1. Sustain implementation |  |  |
| 1. Consider health equity in implementation science |  |  |

1. To what extent did you find the topics useful for your work?

| **Implementation Science Topic** | 1=Not useful  2=Slightly useful  3=Moderately useful  4=Very useful  5=Extremely useful |
| --- | --- |
|  | **Useful for my work** |
| 1. Define an implementation problem |  |
| 1. Conceptualize an implementation problem |  |
| 1. Evaluate barriers to implementation |  |
| 1. Select implementation strategies to overcome the barriers |  |
| 1. Tailor implementation strategies to the context |  |
| 1. Design an implementation study |  |
| 1. Evaluate implementation |  |
| 1. Sustain implementation |  |
| 1. Consider health equity in implementation science |  |

1. What are your thoughts about integrating user-centered design **principles** into the course?

- There was too much user-centered design
- There was the right amount of user-centered design
- I would like more user-centered design in the course
- Other___________________________________

Please explain:

|  |
| --- |

1. What are your thoughts about integrating user-centered design **tools** into the course?

- There was too much Mural
- There was the right amount of Mural
- I would like more Mural in the course
- Other___________________________________

Please explain:

|  |
| --- |

1. What did you think of the guest lectures in Part 1 of the course? (check all that apply)

- They were distracting
- I liked them
- I would like more guest lectures
- There was just the right amount of guest lectures
- There were too many guest lectures
- Other___________________________________

Please explain:

|  |
| --- |

1. Have you applied what you learned in the course? (check all that apply)

- No
- Yes, I apply the conceptual frameworks
- Yes, I use the tools
- Yes, I used the course to help write a paper
- Yes, I use the stakeholder engagement skills
- Yes, I use the user-centered design tools
- Yes, I used the course to help write a grant

1. Thinking back on the course, what could have been improved?

|  |
| --- |
